# Performance Evaluation of Visual Noise Imposed Stochastic Resonance Effect on Brain-Computer Interface Application: A Comparison Between Motion-Reversing Simple Ring and Complex Checkerboard Patterns

**DOI:** 10.3389/fnins.2019.01192

**Published:** 2019-11-08

**Authors:** Jun Xie, Guangjing Du, Guanghua Xu, Xingang Zhao, Peng Fang, Min Li, Guozhi Cao, Guanglin Li, Tao Xue, Yanjun Zhang

**Affiliations:** ^1^School of Mechanical Engineering, Xi’an Jiaotong University, Xi’an, China; ^2^State Key Laboratory of Robotics, Shenyang Institute of Automation, Chinese Academy of Sciences, Shenyang, China; ^3^State Key Laboratory for Manufacturing Systems Engineering, Xi’an Jiaotong University, Xi’an, China; ^4^CAS Key Laboratory of Human-Machine Intelligence-Synergy Systems, Shenzhen Institutes of Advanced Technology, Shenzhen, China

**Keywords:** brain-computer interface (BCI), visual noise, stochastic resonance (SR), motion-reversing stimulation, checkerboard, single ring

## Abstract

Adding noise to a weak input signal can enhance the response of a non-linear system, a phenomenon known as stochastic resonance (SR). SR has been demonstrated in a variety of diverse sensory systems including the visual system, where visual noise enhances human motion perception and detection performance. The SR effect has not been extensively studied in brain-computer interface (BCI) applications. This study compares the performance of BCIs based on SR-influenced steady-state motion visual evoked potentials. Stimulation paradigms were used between a periodically monochromatic motion-reversing simple ring and complex alternating checkerboard stimuli. To induce the SR effect, dynamic visual noise was masked on both the periodic simple and complex stimuli. Offline results showed that the recognition accuracy of different stimulation targets followed an inverted U-shaped function of noise level, which is a hallmark of SR. With the optimal visual noise level, the proposed visual noise masked checkerboard BCI paradigm achieved faster and more stable detection performance due to the noise-enhanced brain responses. This work demonstrates that the SR effect can be employed in BCI applications and can achieve considerable performance improvements.

## Introduction

A brain-computer interface (BCI) is a system that can translate people’s intentions from brain activity into commands to control external devices (Wolpaw et al., 2000). Several electroencephalogram (EEG) signals can be employed to develop BCIs, such as the P300 component of event-related potentials (ERP), slow cortical potentials (SCP), and steady-state visual evoked potentials (SSVEP). Among these, SSVEP is a type of periodic brain response elicited by repetitive visual stimuli at a specific frequency (Muller-Putz and Pfurtscheller, 2008). SSVEP-based BCIs have been widely studied due to their high information transfer rate (ITR) and little required training (Moghimi et al., 2013). However, SSVEP-based BCIs still meet a number of challenges in practical applications. For example, the conventional SSVEP stimulation (e.g., flickering of monochromatic rectangles on a computer screen) is not user friendly and can quickly cause visual fatigue, which leads to poor performance. To alleviate the user’s visual fatigue, BCI paradigms based on motion perception have been proposed in recent years (Heinrich and Bach, 2003). For example, Xie et al. (2012) designed a novel BCI paradigm with motion-reversed Newton’s rings to elicit steady-state motion visual evoked potentials (SSMVEP). This improves the BCI performance and achieves a higher recognition accuracy with less visual discomfort.

Two types of visual stimulation paradigms, i.e., motion-reversing simple single ring stimulus (Xie et al., 2014) and motion-reversing complex alternating checkerboard stimuli (Han et al., 2018), have been used to evoke SSMVEP for BCI applications. In this study, the complex checkerboard was composed of concentric simple rings, all of which shared the same motion-reversing frequency (MRF) as a single ring. The effects of simple and complex stimulation patterns on BCI performance have been investigated in many studies. It has been reported that both complex and simple patterns showed different features when evoking brain responses. Complex stimuli produced stronger brain responses than simple stimuli at the same frequency, which is a clear advantage of complex stimuli over simple stimuli (Vialatte et al., 2010). Lalor et al. (2005) indicated that BCI based on complex visual stimulation enabled improved performance compared to BCI based on simple stimulation. Waytowich et al. (2016) also reported that BCI based on a complex stimulation achieved a higher information transfer rate than BCI based on a simple stimulation under specific conditions. For a complex stimulation, Yan et al. (2017) designed an equal-luminance colored ring-shaped complex checkerboard paradigm that evokes prominent SSMVEP with high signal-to-noise ratios (SNR) in the low-frequency range. These studies show that complex stimuli promotes prominent responses compared to simple stimuli. However, the effect of stochastic resonance (SR) in selecting stimulation patterns remains unclear. This is particularly the case when considering the enhanced SR effects on BCI performance (Xie et al., 2014), on the influence of mental load and fatigue in BCI application (Xie et al., 2017; Zhang and Gao, 2019), as well as on eliciting plasticity in BCI interventions (Xie et al., 2018). To the best of our knowledge, the different influences of the SR mechanism on the performance of motion-reversing complex alternating checkerboard and a simple single ring based BCIs has, to date, not been directly compared.

It is well known that the human brain is intrinsically noisy. As a random fluctuation, noise is widely observed in both the human visual system and other sensory systems (Manjarrez et al., 2007; Faisal et al., 2008). Several studies have demonstrated that the human central nervous system has exploited this inherent neural noise to its structural and functional benefit. Noise was found to contribute to information transfer over axons by increasing the synchronization of neuronal firing (Neiman et al., 1999; Wang et al., 2004). Aihara et al. (2008) have shown that internal noise plays a vital role in enhancing the detectability of weak input signals in the visual system. These phenomena can be explained by the SR mechanism, which was first proposed by Benzi et al. (1981) to explain the periodic recurrence of ice ages. SR is a phenomenon that exists in non-linear systems where the output of a weak periodic input signal can be enhanced by a non-zero level of noise. Moreover, researchers have found that subjects can benefit from SR effects when presented with external visual noise in visual detection tasks, which indicates that the SR effect not only depends on internal but also on external noise (Manjarrez et al., 2002; Long et al., 2004). Experiments showed that the addition of an optimal level of external noise could enhance the detectability of weak input signals, while excessive noise leads to a deterioration of performance (Chatterjee and Oba, 2005). Srebro and Malladi (1999) reported that external noise could induce the SR effect in visually evoked potentials (VEP), which inspired the employment of the SR effect on BCI applications in this study.

A few existing studies utilize the SR effects for BCI applications. Xie et al. (2014) designed a SSMVEP-based BCI by masking visual noise on a motion-reversing ring to achieve better offline and online performance than the simple motion ring stimulation due to the SR effect. The work demonstrated that the addition of visual noise on the motion stimulation could enhance BCI performance. However, only the influence of external noise on the performance of simple stimulation (i.e., motion-reversing single ring stimulation) has been explored to date, while the influence on complex stimulation (i.e., motion-reversing checkerboard stimulation) has not been evaluated. Given that a motion-reversing checkerboard stimulation may perform better than a motion-reversing single ring stimulation, the optimized external visual noise on the motion-reversing checkerboard may achieve superior BCI performance.

Therefore, the present study compares different influences of external visual noise on simple motion-reversing single ring and complex alternating motion-reversing checkerboard stimulation paradigms. To investigate the SR effect on these two types of motion stimuli, visual noise of different intensity levels was used to mask the stimulation. The visual noise intensity level was graded by noise standard deviation (NSD) values of 0, 24, 40, or 56, respectively. First, the characteristics of the brain response under the SR mechanism were examined *via* amplitudes of the evoked SSMVEP. To observe the SR effect of recognition accuracy under different noise intensity levels, canonical correlation analysis (CCA) was introduced to calculate the recognition accuracy within a short stimulation duration. The number of EEG recording channels required for an accurate analysis of both types of stimuli was then compared. Furthermore, time-frequency analysis of these two types of motion stimulations under different intensity levels was implemented, which showed that visual noise could help evoke much stronger SSMVEP in the early stage of stimulation. Finally, the recognition accuracies of both the simple motion-reversing single ring and the complex motion-reversing checkerboard paradigms with different noise intensity levels were compared. This work further corroborated that BCI applications could benefit from the SR effect, and a high-performance BCI paradigm was obtained in this study.

## Materials and Methods

### Subjects

Seven male and three female (aged between 22 and 27 years) graduate students from Xi’an Jiaotong University (Shaanxi, China) were recruited as subjects for this study. All subjects had normal or corrected-to-normal vision and had no history of neurologic and psychiatric disorders. All subjects participated in SSVEP-based BCI experiments before but were naive to the visual noise-masked SSMVEP-based BCI paradigm. The experiment was undertaken in accordance with the recommendations of the Declaration of Helsinki, and each participant provided written informed consent and agreed to participate in this study. The study was approved by the institutional review board of Xi’an Jiaotong University.

### EEG Recordings

**FIGURE 1:**
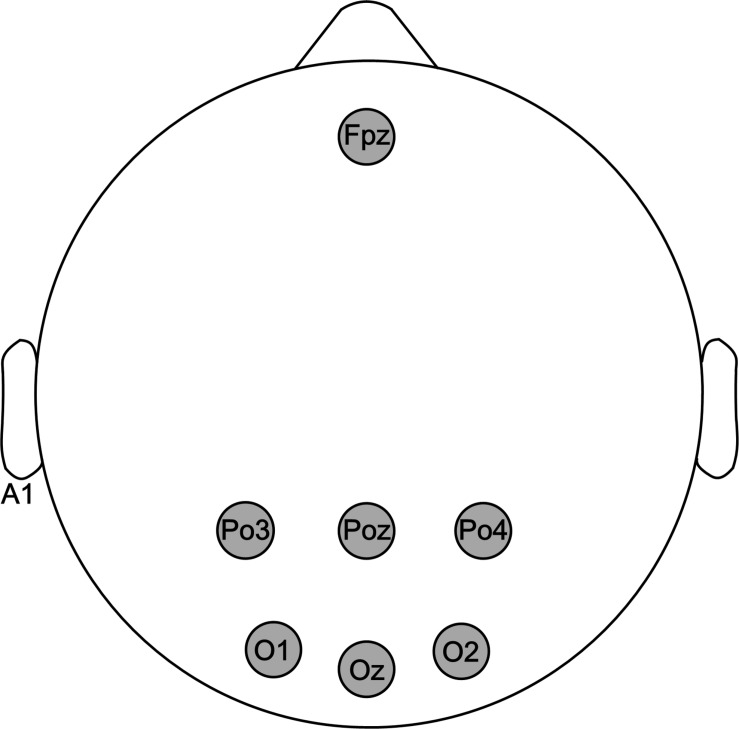
Electrode montage used in this study. EEG signals were recorded from six electrodes: PO3, POz, PO4, O1, Oz, and O2.

The EEG signals were recorded from the occipital region of channels PO3, POz, PO4, O1, Oz, and O2 with a common ground at the frontal position (Fpz) and a reference at unilateral earlobe (A1) according to the international 10–20 system, as shown in Figure 1. A g. USBamp system (g.tec, Graz, Austria) with an Ag/AgCI active electrode system g.GAMMAbox was used to acquire EEG signals at a sampling rate of 1200 Hz. All electrode impedances were retained below 5 kΩ during the experiments.

### Stimulation Design

**FIGURE 2:**
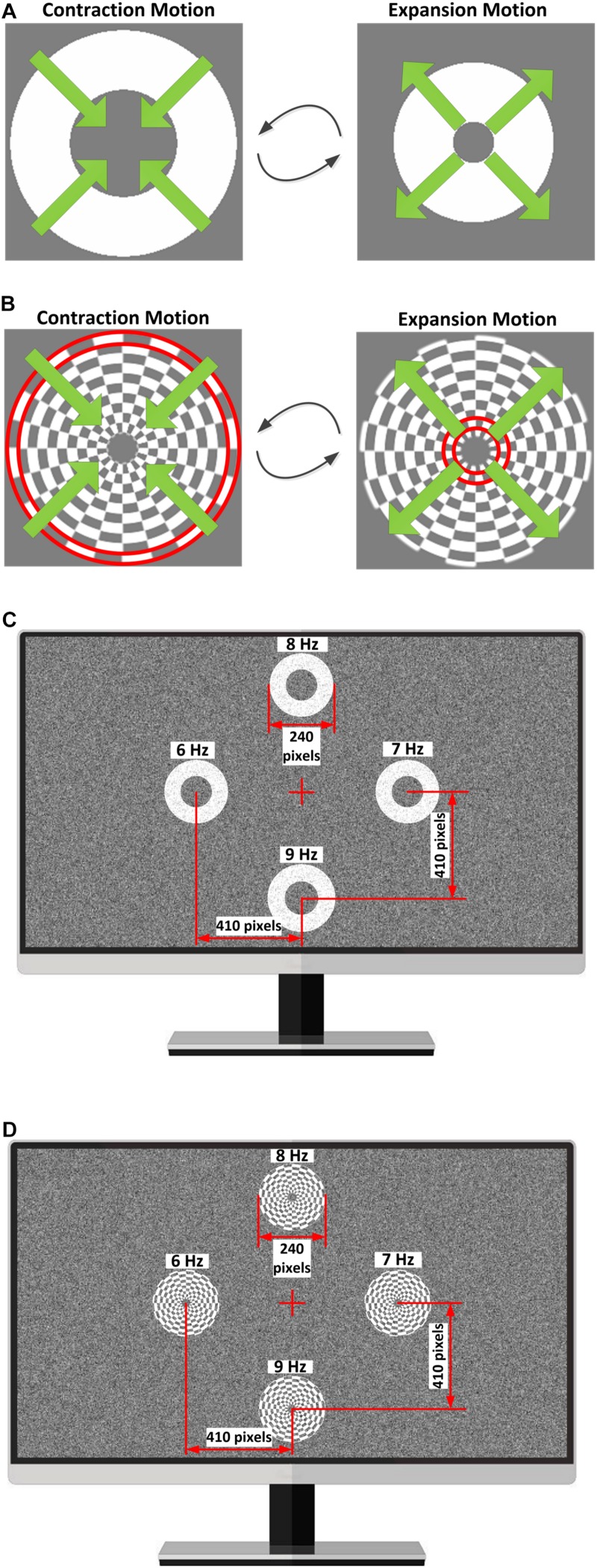
Stimulus paradigms used in this study. **(A)** Contraction-expansion procedure of the motion-reversing single ring under an NSD value of 0. **(B)** Contraction-expansion procedure of the motion-reversing checkerboard under an NSD value of 0. The red boundary line shows a single ring on the checkerboard. **(C)** Distribution of four simple ring stimulators on the screen under an NSD value of 24. **(D)** Distribution of four complex checkerboard stimulators on the screen under an NSD value of 24.

To compare the performance of simple and complex motion paradigms under different noise intensity levels, both simple motion-reversing single ring and complex motion-reversing checkerboard stimuli were designed using the Psychophysics Toolbox Version 3^1^ (Brainard, 1997). As shown in Figure 2A, the width of the motion-reversing single ring remained constant during the expansion-contraction motion procedure. The motion-reversing ring patterns are generated as:

(1) $$I=\left\{ \begin{array}{c} I_{0}+N\,S\,D\cdot r\,a\,n\,d\,n,R_{\varphi }-D<r\,\left(x,y\right)<R_{\varphi }\\ I_{1}+N\,S\,D\cdot r\,a\,n\,d\,n,o\,t\,h\,e\,r \end{array}\right.$$

where *I* represents the luminance of the stimulation, *I*_0_ represents the luminance of constant width of the motion ring with a gray level of 256 (i.e., white color), *I*_1_ represents the luminance of the background with a gray level of 128 (i.e., gray color), the noise intensity level is graded by noise standard deviation (NSD) values of 0, 24, 40, and 56 in this study, *randn* represents a Matlab function that generates random noise with zero mean and unit standard deviation, *r*(*x*, *y*) represents the radius of the pattern pixel points (*x*, *y*), *D* represents the constant width of the motion ring and was set to 60 pixels, *R*_φ_ represents a radius modulated by the sinusoidal function, which controls the contraction and expansion of the motion ring. *R*_φ_ can be defined as:

(2) $$R_{\varphi }=A\cdot \frac{\sin\left(2\,\pi \cdot f_{c}\cdot t\right)+1}{2}+R_{\text{inner}}$$

where *A* represents the amplitude of the expansion-contraction motion process and was set to 60 pixels, *R*_inner_ represents the minimum radius of motion ring and was set to 60 pixels, *f*_*c*_ represents the MRF and was set to 6 Hz, 7 Hz, 8 Hz, and 9 Hz in this study. When the phase of the sinusoidal function shifts from 0 to π, the motion ring contracts and then, expansion of the motion ring is achieved with phase shifting from π to 0. Consequently, the radius of the moving ring ranges from 60 to 120 pixels throughout the contraction-expansion procedure. Figure 2A shows the contraction-expansion procedure of the motion-reversing single ring for an NSD value of 0.

The motion checkerboard pattern under an NSD value of 0 is shown in Figure 2B. The method of constructing motion checkerboard patterns is similar to that for the motion-reversing single ring but more complex. The checkerboard is composed of a series of concentric single rings, each of which is divided into 24 equal grids with an alternative arrangement of gray and white squares. The outer and inner diameters of the motion checkerboard were set to 12 and 120 pixels, respectively. The visual noise used in this study consisted of two-dimensional (2D) spatial-temporal noise speckles that obeyed 2D Gaussian intensity distributions and was updated at a refresh rate of 120 Hz.

In this experiment, a Philips 27-inch LCD screen with a 1,920 × 1,080-pixel resolution (0.31 mm width per pixel) and a 120 Hz refresh rate was used to present the stimulation. Four stimulators were simultaneously displayed on the screen and were distributed in a rhombus layout. The distances of the screen center to the centroid of each stimulator were 410 pixels and were approximately at a 10.4° visual angle when viewed by the subjects at a fixed distance of 0.7 m. The MRFs of the right, left, up, and down stimulators were 6 Hz, 7 Hz, 8 Hz, and 9 Hz, respectively, with a 120 Hz refresh rate. Figures 2C,D show the distribution of the four stimulators on the computer screen under an NSD value of 24.

### Experimental Procedure

**FIGURE 3:**
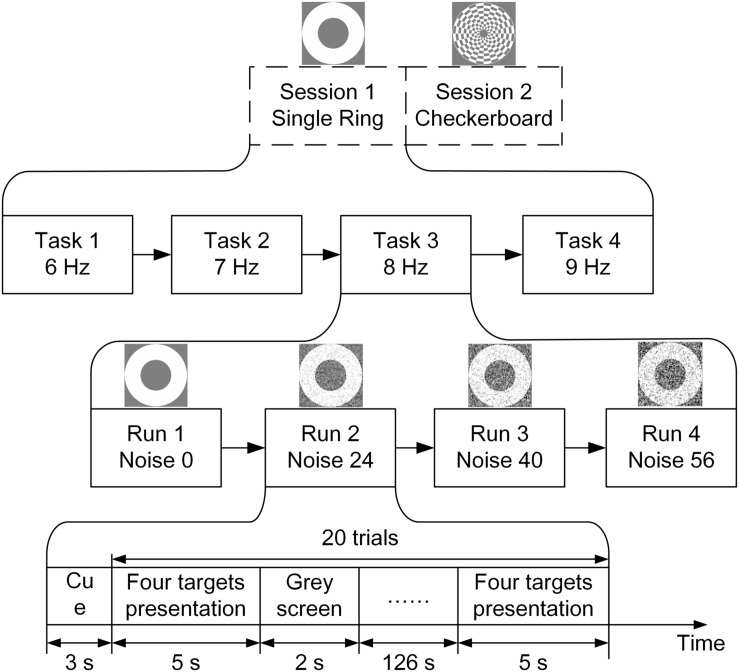
Experimental procedure.

In the offline experiments, each subject participated in two separate sessions. The motion-reversing single ring paradigm was presented in session 1 and the motion-reversing checkerboard paradigm was presented in session 2. Each session was comprised of four tasks corresponding to four different stimulation frequencies (i.e., 6, 7, 8, and 9 Hz). For each task, four runs were conducted with NSD values of 0, 24, 40, and 56. Each run contained 20 trials and each trial lasted for 5 s. The interval between two adjacent trials was fixed at 2 s. Therefore, the time of each single run was about 138 s. Subjects were instructed to focus their attention only on the target stimulator and to avoid any physical movement during each run. A short break was allowed between two runs. Figure 3 shows the experimental procedure. Each experimental session lasted for about 1 h per subject.

An online experiment was proposed to demonstrate the BCI performance of the noise masked motion-reversing checkerboard paradigm. In the online experiments, the subjects were asked to perform two tasks, including checkerboard with noise at NSD value of 40 and checkerboard without noise. The motion-reversing frequency of stimuli was set at 6, 7, 8, and 9 Hz in both the noise-masked and the non-noise task. Each frequency task contains 20 trials and every trial lasts 2 s with a 0.5 s inter-trial interval for gaze shifting.

In our experiments, four targets (their motion frequencies were 6, 7, 8, and 9 Hz) were simultaneously presented on the screen in each trial, and subjects were requested to focus on the specific target in each trial. The recognition accuracy was defined as the percentage of correctly judged trials by the CCA detection method in total trials.

### Preprocessing of the EEG Data

The EEG data collected from each subject was analyzed offline. Band pass filtering from 2 to 45 Hz was performed to remove unwarranted signals. The EEG data segments were extracted from the starting time to the ending time of each trial.

### CCA Analysis

CCA is a multivariable statistical method for the exploration of the underlying correlation between two multivariate sets of variables (Hotelling, 1936). The CCA method has been successfully applied for multi-channel SSVEP detection (Bin et al., 2009). Suppose that there are *K* stimulators with *K* stimulation frequencies *f*_*i*_ (*i* = 1, …, *K*), for the recognition of the target stimulator, two sets of signals should be introduced to CCA. One set of signals is the EEG signals *X*, which are collected from *C* channels with *S* sampling points. A further set of signals is the reference signals *Y*_*i*_, which are constructed at the stimulation frequency *f*_*i*_ and its multi-harmonics:

(3) $$Y_{i}=\left(\begin{array}{c} \cos\left(2\,\pi \cdot f_{i}\cdot t\right)\\ \begin{array}{c} \sin\left(2\,\pi \cdot f_{i}\cdot t\right)\\ \vdots \\ \cos\left(2\,\pi \cdot H\,f_{i}\cdot t\right) \end{array}\\ \sin\left(2\,\pi \cdot H\,f_{i}\cdot t\right) \end{array}\right),t=\frac{1}{F\,s},\cdots ,\frac{S}{F\,s}$$

where *Fs* represents the sampling rate, and *H* represents the number of harmonics. A vector of correlation coefficients [ρ*_*i*_*_1_, ρ*_*i*_*_2_, …, ρ*_*i*_*_*min(C, 2H*__)_] between *X* and *Y*_*i*_ can be obtained by solving the following problem:

(4) $$\max_{W_{x},W_{y_{i}}}\rho \,\left(x,y_{i}\right)=\frac{E\,\left(W_{x}^{T}\,X\,Y_{i}^{T}\,W_{y\,i}\right)}{\sqrt{E\,\left(W_{x}^{T}\,X\,X^{T}\,W_{x}\right)\,E\,\left(W_{y\,i}^{T}\,Y_{i}\,Y_{i}^{T}\,W_{y\,i}\right)}}$$

The maximum of ρ corresponds to the maximum canonical correlation between *X* and *Y*_*i*_. When each canonical correlation of *f*_*i*_ (*i* = 1, …, *K*) was calculated separately, the target could be judged by the maximum ρ of *K* coefficients.

In this study, we use a deformation modality of the CCA method (Valeria et al., 2018). We utilize the top *N* canonical correlation coefficients rather than the maximum one. The top *N* canonical correlation coefficients is combined using the Euclidean norm:

(5) $$r_{i}=\sqrt{\sum_{j=1}^{N}\rho_{i\,j}^{2}}$$

The resulting combination *r*_*i*_ will be regarded as the recognition basis for the stimulation frequency *f*_*i*_. When the combination *r*_*i*_ of each stimulation frequency *f*_*i*_ (*i* = 1, 2, …, *K*) is calculated separately, the target *f*_*target*_ can be assigned to the stimulation frequency with the maximum combination *r*_*i*_:

(6) $$f_{t\,a\,r\,g\,e\,t}=\max_{i=1,\cdots \,K}r_{i}$$

In this study, the stimulation frequency *f*_*i*_ (*i* = 1,…, *K*, *K* = 4) was set to the frequency of each checkerboard or motion ring, the number of channels of *C* was set to 6, the harmonics of *H* was set to 2, and the number of canonical correlation coefficients of *N* was set to 4.

### Statistical Analyses

The values of each individual subject across the non-noise and noise-masked BCI conditions were used by a one-way and three-way analysis of variance (ANOVA) statistic for statistical analysis. To determine the significance, the level of statistical significance was set to *p* < 0.05. The Bonferroni correction was employed in multiple comparisons. The results are expressed as means ± standard deviation (SD).

### Information Transfer Rate (ITR)

Information transfer rate (ITR) is an important criterion to describe the recognition accuracy and the time required by a BCI system. ITR was calculated by:

(7) $$I\,T\,R=\frac{60}{T}\,\left[\log_{2}N+p\,\log_{2}p+\left(1-p\right)\,\log_{2}\left(\frac{1-p}{N-1}\right)\right]$$

Where *T* is the sum of stimulation time of each trial and the time interval between two trials, *N* is the number of targets, and *p* is the average recognition accuracy.

## Results

### SR Phenomena in Both Evoked SSMVEP and Recognition Accuracy

**FIGURE 4:**
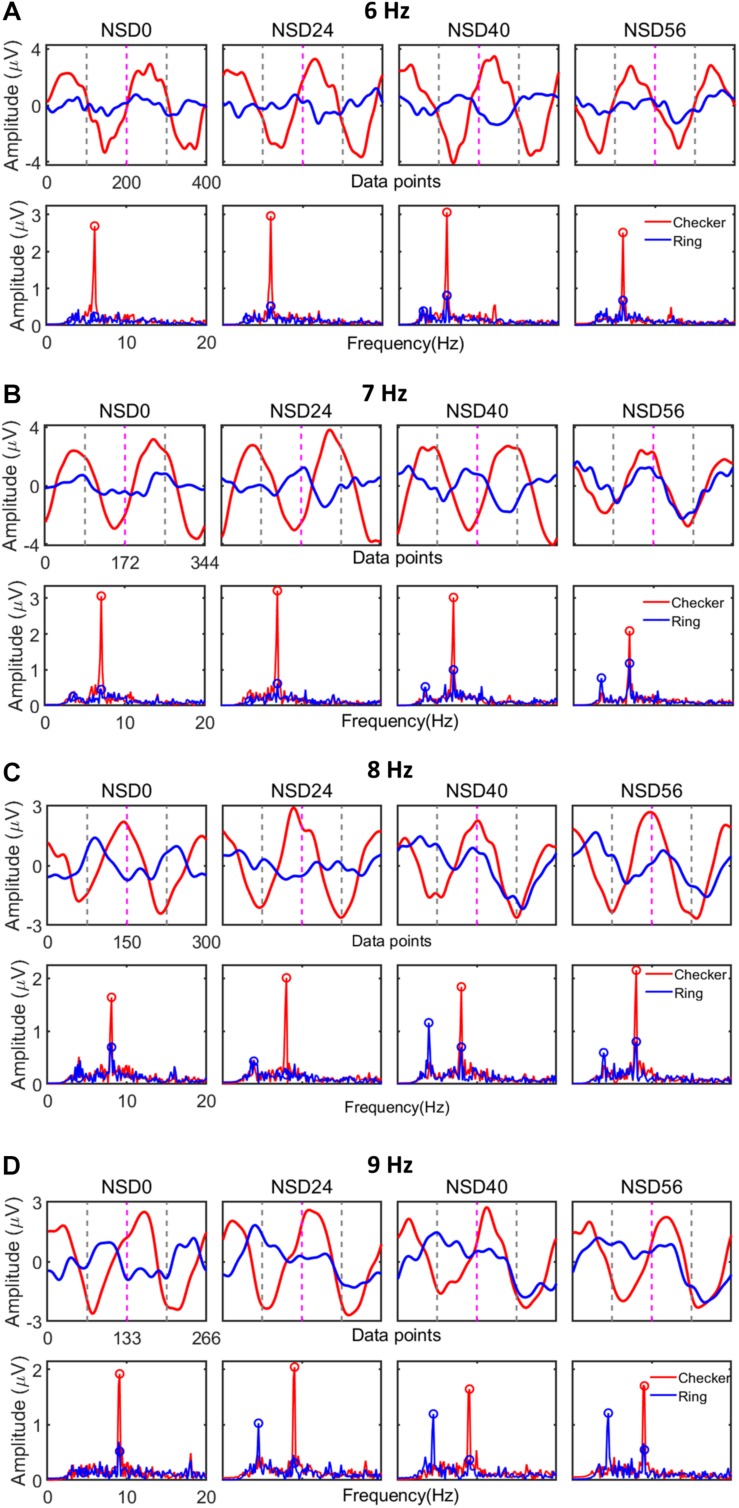
Averaged SSMVEP waveform and corresponding power spectra of Subject S1 at different motion reversion frequencies (MRFs). **(A)** Averaged SSMVEP waveform and power spectra at an MRF of 6 Hz. **(B)** Averaged SSMVEP waveform and power spectra at an MRF of 7 Hz. **(C)** Averaged SSMVEP waveform and power spectra at an MRF of 8 Hz. **(D)** Averaged SSMVEP waveform and power spectra at an MRF of 9 Hz.

Figure 4 shows the averaged EEG waveform and amplitude spectra of Subject S1 from both POz and Oz channels. For this analysis, the averaged EEG waveform was first obtained from the mean value of 20-trial EEG data under the same noise intensity level within the time length of 5 s (i.e., 6000 sampling points). Then, rectangular sliding windows, corresponding to the sample length of two stimulation cycles of the four MRFs (i.e., 400 sampling points for MRF of 6 Hz, 344 sampling points for MRF of 7 Hz, 300 sampling points for MRF of 8 Hz, and 266 sampling points for MRF of 9 Hz), were slid over the averaged EEG data, without overlap, to generate a sequence of data segments. Third, the averaged time-domain waveform of SSMVEP (illustrated in the top row of each panel in Figure 4) was obtained by averaging all data segments under the same noise intensity level. Here, Figure 4 shows the averaged SSMVEP for two stimulation cycles (i.e., four motion reversals) under the noise intensity level marked above. The two stimulation cycles were separated by vertical dotted red lines, and contraction and expansion motion procedures within one stimulation cycle were separated by dotted gray lines. The time-domain EEG waveform in Figure 4 indicates that the amplitude of averaged SSMVEP evoked by 6 Hz motion-reversing checkerboard was progressively enhanced by NSD values of 24 and 40 and diminished with further increasing NSD value. It showed an inverted-U-like resonance shape as a function of the noise intensity level, i.e., the SR characteristic. Similar results were found in motion-reversing checkerboard stimulations at MRFs of 7, 8, and 9 Hz where SSMVEP responses were progressively enhanced by visual noise and peaked in amplitude at an NSD value of 24. The averaged SSMVEP evoked by the motion-reversing single ring also exhibited a similar hallmark of the SR effect. Moreover, the amplitudes of the averaged SSMVEP evoked by motion-reversing checkerboard exceeded those evoked by the motion-reversing single ring. The maxima of averaged SSMVEP magnitudes evoked by the motion-reversing checkerboard at MRFs of 6, 7, 8, and 9 Hz were about two to three times higher than the corresponding single ring paradigm. For the SSMVEP spectra, the averaged EEG data were submitted to a fast Fourier transform (FFT) operation for frequency-domain analysis. The bottom row of each panel in Figure 4 shows the power spectra at NSD values marked above. The power spectra also exhibited an inverted-U-like resonance shape as a function of their noise intensity level. The SSMVEP spectra evoked by motion-reversing checkerboard at MRFs of 6, 7, 8, and 9 Hz increased about two to four times compared to the corresponding single ring stimulation. Interestingly, the checkerboard stimulation evoked fewer harmonic components in SSMVEP spectra than that evoked by the single ring paradigm. This indicates that the SSMVEP evoked by the checkerboard stimulation have the properties of a high energy concentration in the evoking frequency.

**FIGURE 5:**
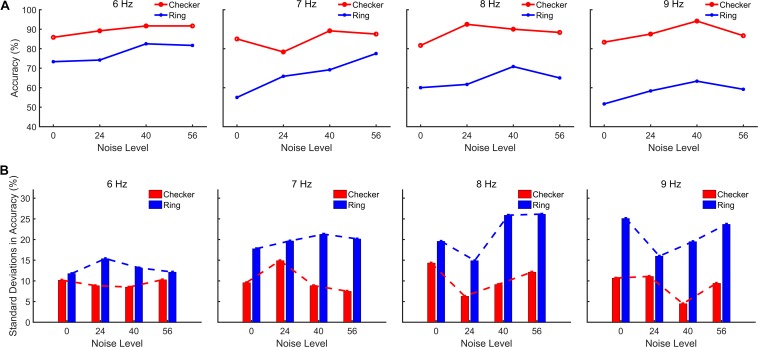
Grand-averaged detection accuracies as a function of NSD values. **(A)** Averaged detection accuracies with a time-window length of 2 s for all subjects. **(B)** Standard deviations of the averaged detection accuracies across subjects.

The EEG data of all subjects recorded from six occipital channels (PO3, POz, PO4, O1, Oz, and O2) were used for CCA-based SSMVEP detection. The recognition accuracy of subjects was evaluated as the percentage of correctly judged trial numbers within each run under a certain noise level. Considering that if the stimulation duration is sufficiently long, the SSMVEP detection accuracy during that time-window length would generally be high (da Cruz et al., 2015), it may be that the exhibit differences between the recognition accuracies of different noise levels are not evident. Therefore, the accuracies of the four-target recognition tasks under a short time-window length (i.e., 2 s adopted for this analysis) were obtained to estimate the visual noise induced SR effect in both motion-reversing checkerboard and single ring paradigms. The top row of Figure 5 shows the averaged recognition accuracies of different noise levels across all subjects with NSD values marked below. The averaged recognition accuracies also exhibited an inverted-U-shaped relationship with the intensity level of visual noise, i.e., all noise-masked stimulations showed a certain degree of enhancement of recognition accuracies compared to stimulation with an NSD value of 0, which is similar to the trend of SSMVEP magnitudes and spectral characteristics. For the checkerboard paradigm, the recognition accuracies at the resonance points of the four MRFs of the noise-masked condition significantly increased by 10.1% (resonance points: 94.5% ± 7.2, non-noise points: 85% ± 12.2; *F* = 8.422, *p* = 0.006) compared to the non-noise condition across all subjects. Furthermore, the non-noise checkerboard paradigm also achieved significantly higher accuracies over the noise-masked single ring paradigm at the resonance points of the four MRFs (for the resonance points of the noise-masked single ring paradigm: 73.5% ± 21.9; *F* = 4.226, *p* = 0.046). This indicates that the noise-masked checkerboard paradigm achieved superior performance in comparison to the other three paradigms. Furthermore, the bottom row of Figure 5 shows that the standard deviations of the average recognition accuracies followed a U-shaped relationship with noise and the resonance points at which the curves in the top row of Figure 5 reached their peaks had the lowest standard deviations. This finding was associated with the hallmark of SR, which is the inverted U-shape relationship between the BCI performance and noise, and the BCI paradigms at the resonance points had a more stable performance with relatively lower standard deviations. Both average accuracies and SSMVEP magnitudes of both types of stimulation exhibited this signature.

### Influence of Selected Channel Number on Recognition Accuracy

**FIGURE 6:**
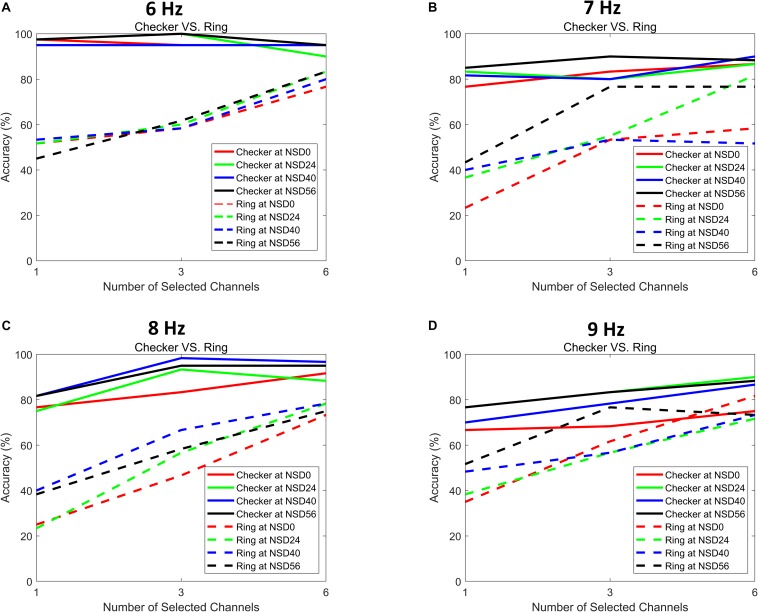
Performance comparisons for different channel selections. **(A)** Averaged detection accuracies of different channel selections at an MRF of 6 Hz. **(B)** Averaged detection accuracies of different channel selections at an MRF of 7 Hz. **(C)** Averaged detection accuracies of different channel selections at an MRF of 8 Hz. **(D)** Averaged detection accuracies of different channel selections at an MRF of 9 Hz.

EEG signals from multiple channels are always required for the CCA detection method. However, employing too many channels may introduce redundant information, which degrades the BCI performance (Arvaneh et al., 2011). To further exploit the advantages of this noise-masked motion checkerboard stimulation, the relationship between the number of selected channels and recognition accuracies was compared. The EEG signals of all subjects were selected for the channel selection analysis. The recognition accuracies of all subjects were calculated with the CCA method from one EEG channel (i.e., Oz), three EEG channels (i.e., O1, Oz, and O2), and six EEG channels (i.e., PO3, POz, PO4, O1, Oz, and O2), respectively. Therefore, for each of the motion-reversing checkerboard and single ring paradigms, different numbers of channels, ranging from one to all six channels, were selected by varying the NSD value. The results shown in Figure 6 indicate that the use of the checkerboard paradigm instead of the single ring paradigm leads better channel selection accuracies, particularly when the number of the selected channels is relatively small. The checkerboard paradigm maintained stable performance when the number of the selected channels varied from one to all six channels. In contrast, the single ring-based paradigm required more channels to achieve higher accuracy compared to the complex checkerboard paradigm, i.e., all accuracy curves of the single ring stimulation followed an increasing trend with an increased number of channels. Specifically, the single ring-based paradigm decreased significantly by an average of 26.3% of the performance in accuracy with a decrease of the channel number from 6 to 3 (*F* = 18.908, *p* < 0.001) and from 3 to 1 (*F* = 28.612, *p* < 0.001) in both non-noise and noise-masked conditions. The noise-masked single ring paradigm at the resonance points provided significantly higher accuracies compared to the non-noise single ring paradigm (*F* = 4.294, *p* = 0.042). Moreover, the improvement in accuracy and the reduction in number of channels were more salient when the complex checkerboard paradigm was applied as compared to the single ring paradigm. On average, the noise-masked checkerboard paradigm, using a single Oz channel at its resonance points, achieved significantly better detection accuracies than the noise-masked single ring paradigm that used all six channels at its resonance points (*F* = 4.547, *p* = 0.047). This suggests that the single channel of EEG signals in the checkerboard paradigm contains sufficient SSMVEP information. Therefore, the CCA method requires fewer EEG channels for recognition in the checkerboard paradigm, while the CCA method requires more EEG channels for the detection of the SSMVEP responses in the single ring paradigm. So, the proposed visual noise masked complex checkerboard paradigm is capable of removing redundant channels in the SSMVEP detection. If fewer electrodes are used in BCI experiments, it could consequently reduce the preparation time and increase the convenience of the BCI application.

### Addition of Visual Noise Promoted the BCI Performance

Since the SSMVEP evoked by stimulation can be enhanced by the SR effect, this study further examined whether BCI performance would benefit from visual noise. To evaluate the BCI performance, both the time-varying characteristics of the brain responses and the recognition accuracies under different time-window lengths were investigated.

**FIGURE 7:**
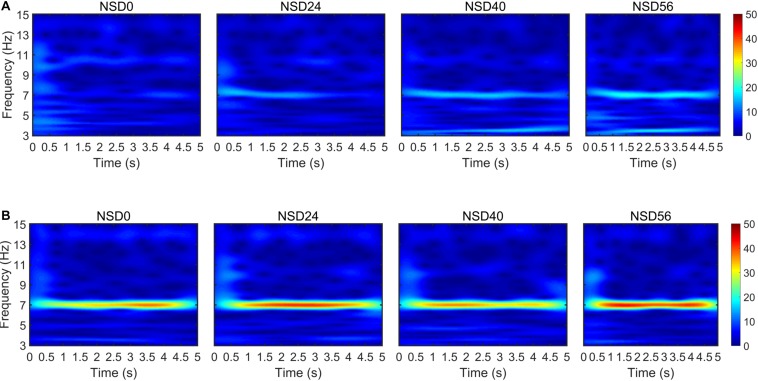
Time-frequency analysis results of SSVMEP signals based on a complex Morlet CWT with a time-window length of 5 s (i.e., 6000 sampling points). **(A)** Time-frequency graph of SSMVEP signals at an MRF of 7 Hz under different NSD values in the single ring paradigm. **(B)** Time-frequency graph of SSMVEP signals at an MRF of 7 Hz under different NSD values in the checkerboard paradigm.

To explore the time-varying characteristics of the brain responses in response to the SR effect, the elicited SSMVEP were analyzed *via* a continuous wavelet transform (CWT). Figure 7 shows a complex Morlet CWT time-frequency analysis of Subject S1 at an MRF of 7 Hz. The top and bottom row of the time-frequency graph shows the results of the time-frequency analysis of the SSMVEP evoked by motion-reversing single ring and checkerboard stimulations, respectively. The time-frequency graph shows that the spectral power in the whole time duration corresponding to the MRF of 7 Hz in the checkerboard stimulation was stronger than that of single ring stimulation. In particular, the motion checkerboard stimulation for NSD values of 24, 40, and 56 evoked pronounced SSMVEP in about 0.5 s after stimulus onset, while the SSMVEP appearance in the non-noise checkerboard stimulation was about 1 s. This indicates that the noise-masked motion-reversing checkerboard stimulation evoked stable SSMVEP responses throughout the stimulation duration. This phenomenon illustrates that the visual noise could help to evoke strong SSMVEP in the early stage of the stimulation. As a result, the frequency components of SSMVEP can be detected within a short time-window length with the help of visual noise, which consequently leads to high performance in BCI recognition tasks. In other words, the required stimulation duration in response to visual noise can be shortened compared to the non-noise stimulation condition.

**FIGURE 8:**
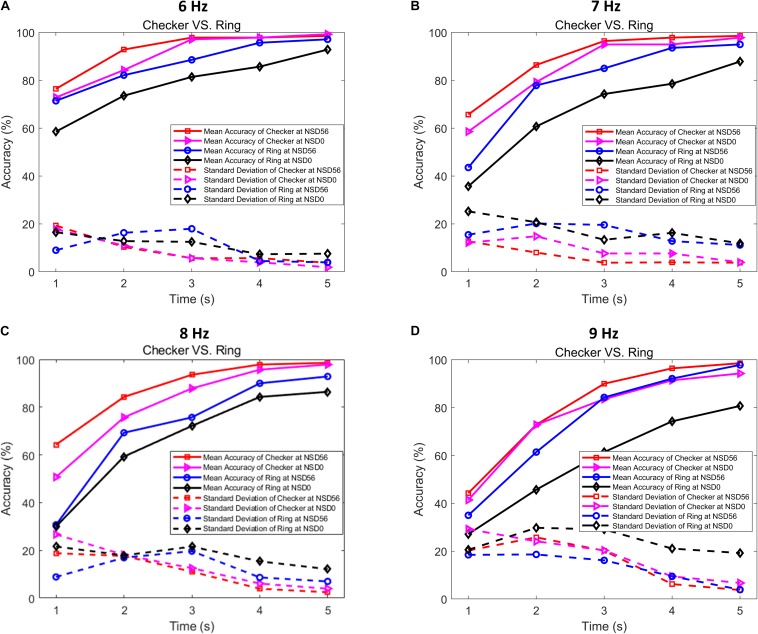
Grand-averaged recognition accuracies and their standard deviations in checkerboard and single ring paradigms with different time-window lengths. **(A)** Averaged recognition accuracies and standard deviations at an MRF of 6 Hz. **(B)** Averaged recognition accuracies and standard deviations at an MRF of 7 Hz. **(C)** Averaged recognition accuracies and standard deviations at an MRF of 8 Hz. **(D)** Averaged recognition accuracies and standard deviations at an MRF of 9 Hz.

To further quantitatively compare the SR effect on the performance of checkerboard and single ring based BCI paradigms, the offline recognition accuracies of both types of stimulations were calculated using the CCA method. Figure 8 depicts the average accuracy across all subjects with time-window lengths from 1 s to 5 s. For the non-noise and noise-masked checkerboard and single ring paradigms, the recognition accuracies showed steady increases with increasing time-window lengths. Overall, the noise-masked checkerboard and single ring stimulation consistently outperformed their corresponding stimulations without visual noise for all time windows from 1 to 5 s. Furthermore, the non-noise checkerboard paradigm also achieved higher accuracies over the noise-masked single ring paradigm. Here, we refer to the checkerboard/single ring patterns as the “stimulation pattern” factor, the MRFs of 6 Hz/7 Hz/8 Hz/9 Hz as the “stimulation frequency” factor, and the noise level of NSD value of 0/56 as the “noise level” factor. Three-way ANOVA test revealed that there are no significant interactions between factors of “stimulation pattern,” “stimulation frequency,” and “noise level” (*p* > 0.05 for all three two-factor interactions). And the above three-way ANOVA test indicated that the noise-masked stimulation pattern provided significantly higher detection accuracies compared to the non-noise pattern for all time window lengths (*p* < 0.001). Most importantly, the noise-masked checkerboard paradigm provided the most significant higher detection accuracies compared to each of the three other paradigms for all four MRFs of all time window lengths (*p* < 0.001 for all time windows, one-way ANOVA with Bonferroni-corrected *post hoc* tests). The average accuracy of the motion-reversing checkerboard stimulation under an NSD value of 56 exceeded 80% for a time-window length of 2 s, and 90% for a time-window length of 3 s, indicating that this noise-masked checkerboard paradigm could achieve a high accuracy at a fast speed. Comparisons of the standard deviation between non-noise and noise-masked checkerboard and single ring paradigms are depicted with dotted lines in Figure 8. The significantly lower standard deviations associated with the noise-masked checkerboard paradigm (*F* = 9.757, *p* < 0.001, one-way ANOVA with Bonferroni-corrected *post hoc* tests) suggest that this paradigm achieves a more stable system performance compared to the three other paradigms. These afore-mentioned results indicate that the motion-reversing checkerboard stimulation with optimized visual noise could provide a more accurate and efficient BCI solution to achieve effective communication with stable recognition performance.

### Online Experiment Results

Table 1 shows the online accuracies and ITRs of the motion-reversing checkerboard paradigm with noise at NSD value of 40 and checkerboard without noise for all subjects. We averaged the accuracies of all subjects for four stimuli frequencies of experimentation. All subjects except S4 exhibited higher recognition accuracies and ITRs in the motion-reversing checkerboard paradigm with noise at NSD value of 40, indicating that the motion-reversing noise masked checkerboard paradigm is a high-performance BCI paradigm.

**TABLE 1:** BCI performance of all subjects in the online experiment.

**Subjects**	**Checkerboard with NSD 40**		**Checkerboard with NSD 0**	
		
	**Accuracy (%)**	**ITR (bits/min)**	**Accuracy (%)**	**ITR (bits/min)**
S1	92.50	35.92	76.25	19.99
S2	92.50	35.92	91.25	34.40
S3	90.00	32.94	83.75	26.45
S4	98.75	45.20	100.00	48
S5	78.75	22.01	73.75	18.08
S6	66.25	13.02	62.50	10.83
S7	75.25	19.21	63.75	11.54
S8	95.00	39.22	78.75	22.01
S9	77.50	20.98	63.75	11.54
S10	97.50	43.00	88.75	31.54
Average	86.50 ± 10.97	30.74 ± 11.10	78.25 ± 12.80	23.44 ± 11.94

## Discussion

Noise is benefit for the signal detection of diverse sensory systems *via* a mechanism known as SR (Douglass et al., 1993; Simonotto et al., 1997; Patrick et al., 2018). However, little research so far utilized the SR mechanism in neural engineering applications. This paper proposes a noise-enhanced BCI paradigm based on a periodic motion checkerboard with dynamic visual noise (see Figure 2B). The evoked brain responses of the noise-enhanced paradigm exhibit an inverted-U shaped relationship with noise intensity, i.e., typical of the SR phenomenon. This provides evidence that visual noise can enhance the detectability of human visual evoked responses. In addition, by combining the SR effect and the advantages of complex motion stimulation, the proposed BCI paradigm evoked much stronger SSMVEP responses and thus achieved a better performance with higher recognition accuracy within a short time window. This suggests that the SR effect has strong potential for BCI applications.

In this study, offline target recognition tasks were implemented with the proposed noise-enhanced paradigm. The experimental results show that the evoked SSMVEP are progressively enhanced by visual noise, up to a maximum point, after which they declined. Moreover, the average recognition accuracies across all subjects for a 2 s duration of both motion-reversing checkerboard and single ring stimulation patterns exhibited an inverted-U shaped pattern as a function of the visual noise level, which is a hallmark feature of the SR phenomenon (Benzi et al., 1981). Nevertheless, the optimal noise intensity at which the curves reached their peaks showed a slight difference. This might be because the intensity of internal noise in living neurons varies widely across subjects and therefore, the intensity of the required optimal external noise differs (Srebro and Malladi, 1999). Despite the slight inconsistency between individual inverted U-shaped curves, the overall SR phenomenon is pronounced. According to previous studies showing that periodic motion stimulation patterns mainly rely on the human perception of motions to elicit the SSMVEP (Xie et al., 2012), the results obtained in this study suggest that external visual noise can enhance the motion perception of the human visual system *via* the SR effect. This is consistent with previous studies where SR enhanced central mechanisms of perception (van der Groen and Wenderoth, 2016). This finding is also consistent with previous studies, which showed that visual noise could improve visual motion perception in a random dot motion (RDM) task (Mario et al., 2016). SR mechanisms in human visual perception might be roughly explained with moderate intensity visual noise assisting sub-threshold periodic stimulation signals to exceed the firing threshold to increase neural firing, and thus to improve the neural signal transmission (Moss et al., 2004). Furthermore, the standard deviations of the average recognition accuracies in the resonance points tended to be the lowest points of the standard deviation curve, implying that the SR effect not only increased the recognition accuracy but also improved the stability of the detection of visual perception.

The motion stimulation can avoid visual fatigue when compared to flicker stimulation (Xie et al., 2012). Similar to the classification of flicker stimulation, the motion stimulation can also be divided into two types, i.e., a complex motion stimulation pattern and a simple motion stimulation pattern (Xie et al., 2014; Han et al., 2018). In this study, the motion-reversing checkerboard was chosen as the complex stimulation pattern. According to previous studies, complex flicker stimulation generates more pronounced SSVEP than simple flicker stimulation (Vialatte et al., 2010); therefore, this study assumed that complex motion stimulation achieves better performance than simple motion stimulation with the enhancement of SR effect. To test this hypothesis, the motion-reversing single ring was chosen as contrast, which is a simple stimulation pattern (see Figure 2A). The offline experimental results showed that the magnitude of the evoked SSMVEP and their Fourier components are higher than that of the single ring stimulation at all stimulation frequencies in both non-noise and noise-masked conditions. These results show that the noise-masked complex motion stimulation pattern elicited much stronger SSMVEP responses than the simple pattern, which extends the findings of previous studies (Lalor et al., 2005). In addition, the performances of both types of motion stimulation patterns were compared if different numbers of channels (ranging from one to six channels) were selected for the CCA method. The recognition accuracies corresponding to each set of selected channels were calculated. According to previous studies, multiple channels might help to increase the recognition accuracy (Bin et al., 2009). However, an excessive number of channels might introduce redundant information, which would not significantly improve the recognition accuracy (Arvaneh et al., 2011). Figure 6 shows that the proposed noise-masked motion checkerboard stimulation pattern requires fewer channels to perform CCA detection because the limited number of channels in this stimulation might already contain sufficient information. These results suggest that the visual noise-masked motion checkerboard stimulation pattern designed in this study offers the advantage of less recording channels needed for target detection, which thus increases the convenience of the BCI application.

The performance of visual noise masked checkerboard paradigm yielded satisfactory results including the speed, accuracy, reliability and other aspects. In this study, a total of 10 subjects participated in the online experiment. Among them, six subjects achieved high accuracy and ITR (>90% / >32 bits⋅min^–1^). This indicated that the visual noise masked checkerboard paradigm can provide excellent performance for the BCI application. For the other four subjects, except S4, though they do not achieve a very high accuracy, their performance also benefited from the noise enhanced effect with accuracy improvements of 15.20% ± 8.16. Additionally, in future work, we will explore other effective classifiers in order to obtain a better performance for the noise enhanced BCI system.

## Conclusion

Combining the SR effect with the advantage of the complex motion stimulation pattern, the proposed noise-enhanced motion checkerboard paradigm achieved a better and more stable performance with regard to speed and accuracy. These experimental results show that the motion checkerboard stimulation, with optimal noise intensity, elicited a stable SSMVEP with a short stimulation duration delay. Moreover, the comparison of the accuracy curves indicates that the proposed noise-enhanced motion checkerboard paradigm has the best performance in the four types of stimulation patterns. This study demonstrates that the SR effect can be utilized in BCI applications where it provides a considerable improvement of BCI performance.

## Data Availability Statement

All datasets generated for this study are included in the article/supplementary material.

## Ethics Statement

The studies involving human participants were reviewed and approved by the institutional review board of Xi’an Jiaotong University. The patients/participants provided their written informed consent to participate in this study.

## Author Contributions

JX conceived of the study, participated in the design of the study, and carried out the experiments. GD carried out the experiments and wrote the manuscript. GX, XZ, and GL designed the study. PF and ML carried out the experiments and corrected the language. GC carried out the statistical analyses. TX collected field data. YZ participated in data analysis. All authors agreed to be accountable for the content of the work.

## Conflict of Interest

The authors declare that the research was conducted in the absence of any commercial or financial relationships that could be construed as a potential conflict of interest.

## Footnotes

1 http://psychtoolbox.org/
